# Systematic review and meta-analysis: does pre-implantation genetic testing for aneuploidy at the blastocyst stage improve live birth rate?

**DOI:** 10.1007/s10815-023-02866-0

**Published:** 2023-07-22

**Authors:** Lorraine S. Kasaven, Diana Marcus, Efstathios Theodorou, Benjamin P. Jones, Srdjan Saso, Roy Naja, Paul Serhal, Jara Ben-Nagi

**Affiliations:** 1 Department of Cancer and Surgery, Imperial College London, Exhibition Rd, South Kensington, London, SW7 2BX UK; 2 Cutrale Perioperative and Ageing Group, Sir Michael Uren Hub, Imperial College London, London, W12 0BZ UK; 3 Department of Gynaecology, Kings College Hospital, Denmark Hill, London, SE5 9RS UK; 4 Centre for Reproductive and Genetic Health, Great Portland Street, London, W1W 5QS UK; 5 Institute for Women’s Health, University College London, 84-86 Chenies Mews, London, WC1E 6HU UK

**Keywords:** Blastocyst, Infertility, Live birth rate, Pre-implantation genetic testing for aneuploidy, Ongoing pregnancy rate, Systematic review, Meta-analysis

## Abstract

**Purpose:**

To establish if preimplantation genetic testing for aneuploidy (PGT-A) at the blastocyst stage improves the composite outcome of live birth rate and ongoing pregnancy rate per embryo transfer compared to conventional morphological assessment.

**Methods:**

A systematic literature search was conducted using PubMed, EMBASE and Cochrane database from 1st March 2000 until 1st March 2022. Studies comparing reproductive outcomes following in vitro fertilisation using comprehensive chromosome screening (CCS) at the blastocyst stage with traditional morphological methods were evaluated.

**Results:**

Of the 1307 citations identified, six randomised control trials (RCTs) and ten cohort studies fulfilled the inclusion criteria. The pooled data identified a benefit between PGT-A and control groups in the composite outcome of live birth rate and ongoing pregnancy per embryo transfer in both the RCT (RR 1.09, 95% CI 1.02–1.16) and cohort studies (RR 1.50, 95% CI 1.28–1.76). Euploid embryos identified by CCS were more likely to be successfully implanted amongst the RCT (RR 1.20, 95% CI 1.10–1.31) and cohort (RR 1.69, 95% CI 1.29–2.21) studies. The rate of miscarriage per clinical pregnancy is also significantly lower when CCS is implemented (RCT: RR 0.73, 95% CI 0.56–0.96 and cohort: RR 0.48, 95% CI 0.32–0.72).

**Conclusions:**

CCS-based PGT-A at the blastocyst biopsy stage increases the composite outcome of live births and ongoing pregnancies per embryo transfer and reduces the rate of miscarriage compared to morphological assessment alone. In view of the limited number of studies included and the variation in methodology between studies, future reviews and analyses are required to confirm these findings.

## Introduction

Despite significant advances within the field of in vitro fertilisation (IVF) and assisted reproductive technologies (ART), the majority of IVF cycles remain unsuccessful with respect to achieving a live birth. Subsequently, embryonic aneuploidy is often the primary reason associated with poor reproductive outcomes, clinically manifested by repetitive implantation failures or recurrent pregnancy loss [1]. This is increasingly common with advancing maternal age, particularly above 37 years old [2, 3]. To mitigate the high failure rate associated with aneuploidy, multiple embryos are often transferred to achieve a single live birth [1]. However, this practice is associated with high multiple pregnancy rates, along with its related obstetric and neonatal burdens [4]. As such, subsequent efforts have since focused on selecting the best quality single embryo for transfer [5].

Traditional methods to assess embryo quality include morphological assessment. However, embryo evaluation at the blastocyst stage cannot accurately predict aneuploidy status, as exemplified by the finding that almost half of the top-quality blastocysts are aneuploid [6]. Further studies have reaffirmed that traditional methods of morphologic embryo selection are unable to detect aneuploidy reliably [7]. Since 1993, pre-implantation genetic testing for aneuploidy (PGT-A), previously termed pre-implantation genetic screening (PGS) however, has utilised a number of methodologies for genetic testing to overcome such challenges [8]. The procedure offers an opportunity to screen embryos for certain chromosomal abnormalities in order to prioritise embryos with euploid (putative diploid) test results for transfer, thereby improving IVF outcomes [9].

Previously, fluorescence in situ hybridisation (FISH) was the most frequently adopted technique used for PGT-A, following blastomere biopsy of cleavage stage embryos. However, a number of studies failed to show any benefit in the live birth rate, especially amongst older women [10–12]. Furthermore, the mosaic nature of cleavage stage embryos, in addition to the ability to only screen a limited number of chromosomes, contributed to the poor initial outcomes following PGT-A using FISH and thus the decline in implementation of this technique [13]. The evolution of genetic testing techniques nonetheless has enabled methods such as comprehensive chromosomal screening (CCS), which entails the analysis of all the chromosomes, offering a much greater degree of utility when compared to FISH techniques [14]. CCS can be performed using array comparative genomic hybridisation (CGH), single nucleotide polymorphism (SNP) arrays, quantitative polymerase chain reaction (qPCR) and next-generation sequencing (NGS). CCS can also be undertaken on biopsies taken at different stages of embryo development, including day 1 zygote (polar bodies), day 3 cleavage-stage (1 or 2 blastomeres), or day 5 or 6 blastocyst stage embryos (3–10 trophectoderm cells). Biopsy of the trophectoderm is deemed to be less traumatic and associated with a lower rate of mosaicism, when compared with biopsy of the blastomere during the cleavage stage [14]. Consequently, biopsy at the blastocyst stage is the most commonly used approach, with NGS as a method of PGT-A.

Despite the theoretically beneficial reproductive outcomes following PGT-A, evidence in favour of such methods remain variable and contradictory [15]. This is despite a number of double-blinded randomised control trials (RCTs) assessing the use of aneuploidy in all 24 chromosomes, from both single and multiple centres included in metanalyses, with overall inconsistent conclusions drawn [11, 12, 16]. The Human Fertilisation and Embryology Authority (HFEA), the UK regulatory body for ART, refers to a traffic light system to rate various add-on treatments, to describe whether the treatments are considered effective at improving the chances of having a livebirth. Green-rated add-ons have been proven by more than one high-quality randomised controlled trial (RCT); amber is rated when evidence from RCTs is conflicting, and red when no evidence from RCTs has been established. As such, the HFEA still stipulates that there is no evidence to suggest that PGT-A on a day 5 embryo is effective and safe [17]. A study assessing the outcomes of PGT-A and non-PGT-A cycles between 2016 and 2018 taken from the HFEA however, has since challenged the HFEA red traffic light guidance, by demonstrating the significant benefit of PGT-A compared to morphology [8]. The study however has been rebutted by various authors who have also analysed HFEA data taken from the same period [18, 19]. In one particular study comparing all PGT-A cycles to a control group, including those that could have had PGT-A had the option been available, the treatment effect of PGT-A was different, with an overall odds ratio (OR) for a live birth event quoted as 0.82 (0.68–1.00) using > 1 transferrable embryo control and 0.80 (0.64–0.99) when using > 5 embryos created as controls [18]. Thus, the analysis demonstrated an overall reduction in live birth rates following PGT-A when comparing like-for-like groups [18]. A separate study utilised data from 7 individual clinics reporting at least 50 PGT-A cycles compared to IVF/intracytoplasmic sperm insemination (ICSI) frozen cycles, taken from the same period as the 2016–2018 HFEA report [19]. The study demonstrated that PGT-A had a potential benefit in differentiating between a viable and non-viable embryo to reduce the risks of clinical pregnancy loss in women > 35 years old (1.97 (1.82–2.12) ≥ 35 years vs 1.12 (1.01–1.24) < 35 years) [19]. The risk ratios for pregnancy losses from clinical pregnancies were similar between clinics, with PGT-A also being favoured in older women (0.51 (0.39–0.68) ≥ 35 years vs 1.09 (0.78–1.54) < 35 years) [19].

Remarkably, the HFEA annual report on IVF trends and figures for 2017 and 2018 did not provide any outcome data for PGT-A. Given that clinicians in the UK are guided by limited and conflicting evidence [20, 21], the effectiveness of PGT-A is not well understood and further studies are required to improve understanding of the reproductive outcomes.

The primary aim of this manuscript is to perform an up-to-date systematic review and meta-analysis to evaluate whether LBR and ongoing pregnancy rates (OPR) per embryo transfer improve with CCS-based PGT-A at the blastocyst stage, when compared to conventional morphological assessment.

## Materials and methods

### Search strategy

During the undertaking of this review, PRISMA guidelines were adhered to [22]. Electronic searches of studies conducted from 1st March 2000 to 1st March 2022 were performed using PubMed, EMBASE and the Cochrane database. The search was limited to studies conducted in humans and written in the English language. Case studies, commentaries, reviews and editorials were excluded. The following search terms were used: (preimplantation genetic screening, PGS, preimplantation genetic testing, PGT-A, PGT, comprehensive chromosome screening, CCS, comparative genomic hybridisation, CGH, array comparative genomic hybridisation, aCGH, single nucleotide polymorphism, SNP, polymerase chain reaction, PCR and next generation sequencing, NGS). All article abstracts were reviewed for relevance, with subsequent reference lists and bibliographies of included studies examined. Furthermore, a manual search of published cases was performed to identify any other relevant cases. The study was not registered and a review protocol was not prepared.

### Study selection and data extraction

Following the removal of duplicate publications, three authors (LSK, ET, DM) independently examined the electronic search results, checking the titles, abstracts and full text for further detail. Any disagreements were resolved by a fourth author (JBN).

Published trials eligible for inclusion included observational or randomised studies comparing women undergoing IVF with PGT-A using CCS technology (any type) and trophectoderm biopsy at the blastocyst stage (defined as CCS group), to women undergoing IVF with standard care without PGT-A (control group). Studies performing CCS at cleavage or polar body stage were excluded. There was no distinction made between the type of CCS technology (NGS, CGH, SNP, qPCR) used or between fresh and frozen cycles. Studies that did not report LBR or OPR were excluded.

### Quality assessment

All studies were assessed for quality using predetermined criteria based on the Newcastle–Ottawa Scale (NOS) [23] and Cochrane handbook for observational and cohort studies [24] by two authors (LK, ET). The NOS looks at 3 different metrics: selection, comparability and outcome. A maximum of 2–4 points are awarded depending on the category. Total higher scores (7 or more) equate to higher quality. The RCTs were assessed for quality using the following criteria: random sequence generation, allocation concealment, blinding of participants and personnel, incomplete outcome data, selective outcome reporting and other potential sources of bias, such as selective reporting of subgroups or potential influence from funders.

### Main outcomes

Amongst the studies included, large heterogeneity regarding the outcome measures and definitions evaluating PGT-A was observed. In this meta-analysis, a composite outcome of LBR and OPR per embryo transfer was the primary outcome measure, as most pregnancies beyond 20 weeks go on to achieve live birth [25]. In addition, the delivery rate was considered synonymous with LBR. Secondary outcomes included implantation rate (IR), miscarriage rate and multiple pregnancy rate.

Biochemical pregnancies were defined as a positive serum b-hCG level (> 5 MIU/mL) without ultrasound confirmation of a gestational sac. A clinical pregnancy was defined as the presence of an intrauterine gestational sac with a viable foetal pole on ultrasound. Implantation rate was defined as the number of intrauterine gestational sacs / total number of embryos transferred per patient. The clinical miscarriage rate was defined as the number of miscarriages divided by the number of clinical pregnancies. A miscarriage was diagnosed only after confirmation of a clinical pregnancy. The multiple pregnancy rate was also analysed, when available. Recurrent implantation failure (RIF) was defined as ≥ 3 failed IVF cycles, despite the transfer of high-quality embryos. Recurrent miscarriage (RM) was defined as ≥ 2 idiopathic miscarriages.

### Statistical analysis

The effect of PGT-A versus non-PGT-A on each outcome measure was analysed separately, where *p* < 0.05 was considered statistically significant. Pooled estimates of risk ratios (RRs) with their 95% confidence intervals were calculated for each study according to a fixed-effects model. The RCTs and cohort studies were analysed separately to minimise selection bias. Statistical heterogeneity amongst the studies was examined by assessing the scatter in the data points and checking for overlap in confidence intervals. In addition, it was tested formally using both the Cochran’s *Q* test and the *I*^2^ index. The Higgins study suggests low, moderate and high heterogeneity corresponding to *I*^2^ values of 25%, 50% and 75% respectively [26]. When heterogeneity was considered high (> 75%), a random-effect model was used to combine the results; otherwise, a fixed-effect model was used. Review Manager 5.4 (Revman version 5.4; Nordic Cochrane Center) was used to combine data and perform the statistical analysis. Forest plots were created for comparison.

## Results

### Literature search and study selection

After the initial search, 1307 studies were retrieved and abstracts were subsequently reviewed. Thirty-seven studies were selected for detailed assessment. The main reasons for exclusion were the following: lack of control group (*n* = 4), biopsies performed at the cleavage or polar body stage or embryo transfers performed on day 3 (*n* = 13) and the LBR or OPR not documented (*n* = 2). A further 2 studies were excluded, as the women in the study were carriers of chromosomal rearrangements. From the selected studies, five stated their exclusion criteria included either single gene diagnosis cycles [27–29], abnormal chromosomes in either or both partners [30] or a plan to undergo PGT-A for monogenic disease or parental chromosomal structural rearrangements [31]. The remaining studies in this meta-analysis did not document whether cycles for chromosomal rearrangements had been excluded or not.

**Fig. 1:**
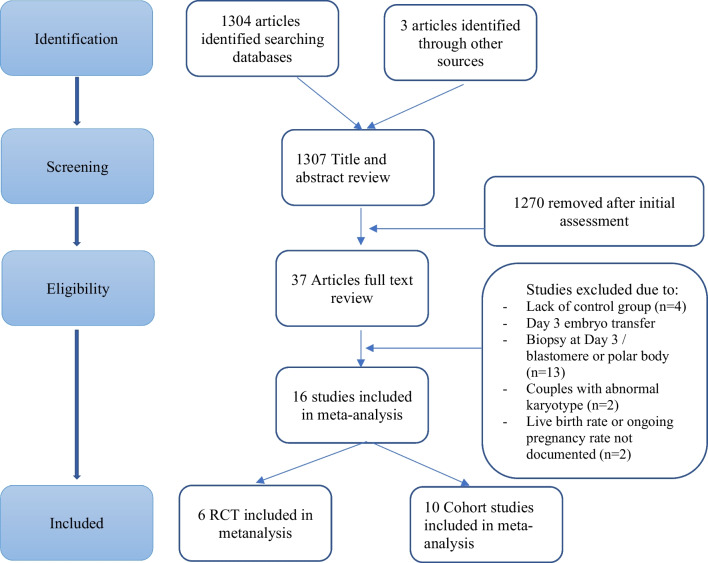
Flow chart of study selection

Finally, six RCTs [25, 31–35] and 10 cohort studies [27–30, 36–41] that assessed LBR and/or OPR per embryo transfer in both PGT-A and non-PGT-A groups fulfilled the inclusion criteria and thus included in the meta-analysis. It is worth noting that the study by Forman et al. was a randomised non-inferiority trial, to determine if the disadvantage of single embryo transfer (SET) relative to double embryo transfer (DET) could be overcome by PGT-A [33]. The study selection process is summarised in Fig. 1.

### Trial characteristics

The main characteristics of the six RCTs and 10 cohort studies are displayed in Tables 1 and 2 respectively. For each study, the design, indication for PGT-A, embryo biopsy stage, CCS platform used, type of embryo transfer (fresh/frozen) and main outcomes are presented. Overall, the 16 included studies accounted for 5793 ART cycles in women ranging from 20 to 43 years old. Of these studies, 8 were performed in America [27–29, 33, 34, 36–38], four were based in Europe, [31, 35, 39, 40], two were undertaken in Asia [30, 41] and two included data from multiple centres worldwide [25, 32]. Over half the studies did not require a formal indication for PGT-A such as the following: advanced maternal age (AMA), RIF or RM. Indeed, the two largest RCTs predominantly included women with good prognosis: less than two prior failed IVF attempts with a good ovarian reserve and at least two–three high-quality blastocysts available [25, 31]. Amongst the RCTs, qPCR was the most common platform utilised to perform CCS, whilst aCGH accounted for the most frequently used in the cohort studies. Indications for undertaking PGT-A included AMA [27, 38, 41], RIF [30, 41] and RM [30, 41] whereas several studies cited no specific indications for PGT-A [29, 31, 35, 39].

**Table 1:** Characteristics of included RCTs

Study (year)	Patients (*n)* (PGT-A vs Control)	Cycles (*n*) (PGT-A vs Control)	Study design	PGT-A (type)	Mean No. ET (PGT-A vs Control)	Fresh or frozen ET	Inclusion criteria	Exclusion criteria	ART and ET	Biopsy	Outcomes
Forman (2013)	*n* = 175 (89 vs 86)	*n* = 175 (89 vs 86)	RCT *(non- inferiority)*	PCR	PGT-A: SET Control: DET	Fresh *n* = 123 Frozen *n* = 52	◦ ≤ 42 years old ◦ One or less failed IVF cycles ◦ Normal uterine cavity ◦ Normal ovarian reserve (AMH ≥ 1.2 ng/mL, day 3 FSH < 12 IU/L) ◦ At least 2 expanded blastocysts suitable for transfer or cryopreservation by day 6 of development	◦ Severe male factor infertility requiring surgical sperm extraction ◦ PCOS ◦ BMI > 30 kg/m^2^ ◦ < 2 expanded blastocysts on day 5	ICSI	Blastocyst Day 5	◦ Ongoing PR ≥ 24 weeks ◦ Multiple pregnancy rate ◦ IR ◦ Clinical miscarriage
Munne (2019)	*n* = 661 (330 vs 331)	*n* = 587 (274 vs 313)	RCT *(prospective)*	NGS	PGT-A: SET Control: SET	Frozen *n* = 661	◦ 25–40 years old ◦ IVF with autologous oocytes ◦ At least two blastocysts of sufficient quality for biopsy ◦ Vitrification by day 6	◦ Diminished ovarian reserve ◦ Previous failed IVF-ETs ◦ > 1 miscarriage ◦ Azoospermia or severe oligospermia ◦ Failure to achieve at least 2 blastocysts by day 5 or 6	IVF	Blastocyst Day 5	◦ Ongoing PR > 20 weeks gestation ◦ Biochemical pregnancy ◦ Miscarriage rate
Ozgur (2019)	*n* = 220 (109 vs 111)	*n* = 191 (80 vs 111)	RCT *(prospective)*	NGS	PGT-A: SET Control: SET	Frozen *n* = 220	◦ ≤ 35 years old ◦ AFC ≥ 5 ◦ BMI ≥ 18 but ≤ 35 kg/m^2^ ◦ 2 blastocysts with scores of ≥ 2BB on day 5 of in vitro embryo development ◦ > 7 mm endometrial thickness and ≤ 2-ng/mL progesterone day 14 of cycle	◦ Any intrauterine and or endometrial abnormalities	ICSI (freeze all cycles)	Blastocyst Day 5	◦ LB > 20 weeks gestation ◦ CPR ◦ Miscarriage rate
Scott (2013)	*n* = 155 (72 vs 83)	*n* = 155 (72 vs 83)	RCT *(prospective)*	qPCR	PGT-A: 1.86 Control: 2.0	Fresh *n* = 155	◦ 21–42 years ◦ No more than one prior failed IVF retrievals ◦ Normal endometrial cavity ◦ Basal FSH level ≤ 15 IU/L ◦ Basal AFC of ≥ 8 ◦ Available ejaculated sperm from male partner ◦ Willing to limit transfer order to a maximum 2 embryos	◦ Less than 2 blastocysts by day 5	ICSI	Blastocyst Day 5	◦ Sustained IR ◦ Chemical and clinical pregnancy rate ◦ Delivery rate
Yan (2021)	*n* = 1212 (606 vs 606)	*n* = 1212 (606 vs 606)	RCT *(non-inferiority)*	NGS	PGT-A: SET Control: SET	Frozen *n* = 1212	◦ 20–37 years ◦ Subfertility and undergoing first IVF cycle ◦ Availability of 3 or more good quality blastocyst (Gardner score 4BC or better)	◦ Uterine abnormality (e.g. uterine congenital malformation, untreated uterine septum, adenomyosis, submucous myoma, endometrial polyps, intrauterine adhesions) ◦ Contraindication to pregnancy ◦ Plan to undergo preimplantation genetic testing for monogenic disease or parental chromosomal structural rearrangements ◦ Use of donated oocytes or sperm	ICSI	Blastocyst Day 5	◦ Cumulative LBR resulting from up to three ETs performed within 1 year after randomisation ◦ LBR ≥ 37 weeks gestation ◦ Birth weight 2500-4000 g ◦ No major congenital anomaly ◦ Cumulative biochemical and CPR ◦ Pregnancy loss rate ◦ Multiple pregnancy rate ◦ Duration of pregnancy ◦ Cumulative incidence of maternal and neonatal complication ◦ Number of ETs needed to achieve LB
Yang (2012)	*n* = 103 (55 vs 48)	*n* = 103 (55 vs 48)	RCT *(Blind)*	aCGH	PGT-A: SET Control: SET	Fresh *n* = 103	◦ Regular ovulation ◦ Female < 35 years old ◦ No prior IVF ◦ Normal intrauterine contour (confirmed on hysteroscopy), intact ovaries ◦ FSH < 10 IU/l on day 2–3 ◦ E_2_ < 60 pg/ml on day 2–3 ◦ Aetiology if infertility included: tubal factor and, or male factor	◦ IVF treatment including donor gametes or frozen/thawed embryos	IVF/ICSI	Blastocyst Day 5	◦ Ongoing PR ≥ 20 weeks gestation ◦ CPR ◦ Multiple pregnancy rate

Key:

*AMH*, anti-mullerian hormone; *aCGH*, microarray-based comparative genomic hybridization; *AFC*, antral follicle count; *BMI*, body mass index; *CPR*, clinical pregnancy rate; *DET*, double embryo transfer; *E2*, oestrogen; *ET*, embryo transfer; *FSH*, follicle stimulating hormone; *ICSI*, intracytoplasmic sperm injection; *IR*, implantation rate; *IVF*, in vitro fertilisation; *LBR*, livebirth rate; *NGS*, next generation sequencing; *PCOS*, polycystic ovarian syndrome; *PCR*, polymerase chain reaction; *PR*, pregnancy rate; *qPCR*, quantitative polymerase chain reaction; *RCT*, randomised controlled trial; SET, single embryo transfer

**Table 2:** Characteristics of included cohort studies

Study (year)	Patients (*n*) (PGT-A vs Control)	Cycles (*n*) (PGT-A vs Control)	Study design	PGT-A (type)	Mean No. ET (PGT-A vs Control)	Fresh or frozen ET (*N* = cycles)	Inclusion criteria	Exclusion criteria	ART and ET	Biopsy	Outcomes
Coates (2017)	*n* = 397 (294 vs 103)	*n* = 398 (294 vs 104)	Retrospective observational cohort	aCGH or NGS	PGT-A: SET or DET Control: SET or DET	Frozen *n* = 397	◦ Oocyte recipients – own uterus or gestational carriers ◦ Caucasian Donor oocytes	◦ ND	IVF	Blastocyst Day 5	◦ LBR per cycle ◦ Live birth per implantation rate (babies born per ET) ◦ Ongoing pregnancy rate ◦ Multiple pregnancy rate
Forman (2012)	*n* = 322 (140 vs 182)	na	Retrospective cohort	Rapid PCR	PGT-A: SET Control: SET	Fresh *n* = not documented Frozen *n* = not documented	◦ AMA (> 35) ◦ Previous failed IVF cycle ◦ History of recurrent pregnancy loss ◦ Couple request	◦ Donor oocytes ◦ PGD for single gene defects ◦ Previously frozen embryos thawed for rapid PCR and same-day transfer	ICSI	Blastocyst Day 5	◦ Ongoing pregnancy rate ◦ Chemical pregnancy rate ◦ Clinical miscarriage rate ◦ Multiple pregnancy rate
Kang (2016)	*n* = 1052 (159 vs 893)	*n* = 1137 (274 vs 863)	Retrospective cohort	aCGH or SNP	PGT-A: SET or DET Control: SET or DET	Fresh *n* = 893 Frozen *n* = 159	◦ All cycles in which one or more embryos underwent biopsy for 24 chromosome screening ◦ All patients with a fresh blastocyst transfer	◦ Severe male factor infertility ◦ PGD for single gene defects	ND	Blastocyst Day 5 or 6	◦ IR ◦ Clinical intrauterine gestation ◦ Miscarriage rate ◦ Biochemical pregnancy rate ◦ LBR
Lee (2015)	*n* = 393 (49 vs 344)	*n* = 620 (170 vs 450)	Retrospective cohort	aCGH	PGT-A:1.14 Control (fresh): 2.67	Fresh *n* = 344 Frozen *n* = 49	◦ Age 40–43 years ◦ Women using their own oocytes	◦ Donor oocytes	IVF and ICSI	Blastocyst Day 5	◦ IR ◦ CPR ◦ LBR per ET ◦ Miscarriage
Lee (2019)	AMA *n* = 122 (61 vs 61)	PGT-A: AMA 87, RIF 82, RM 82, OD 45 Control: AMA 61	Retrospective observational cohort	aCGH	PGT-A: 1.6 ± 0.5 Control: 2.3 ± 0.6	Frozen *n* = 122	◦ AMA (> 38) ◦ RIF (3 or more failed cycles of good embryos) ◦ Idiopathic recurrent miscarriage (> 2 or more) ◦ Oocyte donors	◦ ND	IVF	Blastocyst Day 5 or 6	◦ IR ◦ CPR ◦ Miscarriage rate ◦ LBR
Liss (2018)	*n* = 181 (96 vs 85)	*n* = 197 (112 vs 85)	Prospective cohort	NGS	PGT-A per ET: 1.4 ± 0.5 Control per ET: 1.4 ± 0.5	Frozen n = 181	◦ 1^st^ or 2^nd^ cycle of IVF only	◦ Endometriosis/ Adenomyosis	ICSI/IVF	Blastocyst	◦ LBR per cycle ◦ CPR ◦ Biochemical pregnancy rate ◦ Implantation rate ◦ Miscarriage rate per cycle
Sato (2019)	*n* = 123 (45 vs 78)	*n* = 171 (83 vs 88)	Multicentre prospective pilot	aCGH	PGT-A: SET Control: SET	Frozen *n* = 123	◦ RPL (no previous livebirth, 2 or more miscarriage, where at least one miscarriage was caused by embryonic aneuploidy and pregnancies were following IVF and ET) ◦ RIF (3 or more failed IVF-ET)	◦ Abnormal chromosome in either or both partners ◦ Congenital uterine anomaly ◦ Anti-phospholipid syndrome ◦ Azoospermia	ICSI	Blastocyst Day 5 or 6	◦ LBR ◦ CPR ◦ Biochemical pregnancy loss ◦ Miscarriage rate
Schoolcraft (2010)	*n* = 157 (44 vs 113)	*n* = 158 (45 vs 113)	Prospective matched cohort	CGH	PGT-A: 2.0 Control: 2.7	Frozen *n* = 157	◦ AMA (> 35) ◦ And/or history of unsuccessful IVF treatments or previous spontaneous abortion	◦ ND	ICSI	Blastocyst Day 5	◦ Biochemical pregnancy rate ◦ IR ◦ LBR
Schoolcraft (2013)	*n* = 737 (347 vs 390)	na	Prospective unmatched cohort	SNP	PGT-A: SET Control: SET	Fresh *n* = 118 Frozen *n* = 619	◦ ND	◦ ND	IVF	Blastocyst	◦ IR ◦ Ongoing pregnancy rate ◦ Miscarriage rate
Whitney (2016)	*n* = 287 (134 vs 153)	*n* = 332 (172 vs 160)	Observational retrospective cohort	aCGH	PGT-A: 1.0–1.4. Control: 1.9–2.7	Fresh *n* = 149 Frozen *n* = 138	◦ Only the patients’ first transfer attempt was analysed ◦ Fair to excellent quality blastocysts (≥ 3BB)	◦ Patients who had undergone previous transfers ◦ All patients’ who underwent oocyte retrieval during the timeframe of the study, but did not undergo embryo transfer due to spontaneous pregnancy ◦ Single gene diagnosis cycles and/or oocyte cryopreservation cycles ◦ All PGS cases initiated during the timeframe of the ‘no PGS’ group	IVF ICSI	Blastocyst Day 5 or 6	◦ CPR ◦ IR ◦ LBR

Key:

*aCGH*, microarray-based comparative genomic hybridization; *AMA*, advanced maternal age; *CPR*, clinical pregnancy rate; *DET*, double embryo transfer; *ET*, embryo transfer; *FET*, frozen embryo transfer; *IR*, implantation rate; *ICSI*, intracytoplasmic sperm injection; *IVF*, in vitro fertilisation; *LBR*, livebirth rate; *NGS*, next generation sequencing; *ND*, no data; *NS*, not specified; *PCR*, polymerase chain reaction; *PGD*, preimplantation genetic diagnosis; *RIF*, recurrent implantation failure; *RPL*, recurrent pregnancy loss*; SET,* single embryo transfer*; SNP*, single nucleotide polymorphism;

### Quality assessment

Amongst the 6 RCTs, the study design and quality varied. All studies used a random number generator or function [25, 31–35]. Of these, three used block randomisation [31, 33, 34], with one using separate randomisation tables for each maternal age group [34]. One study randomised patients using an electronic data capture system and randomisation module according to patient age group [25]. The method of allocation and concealment was described explicitly amongst three studies only [31, 33, 34]. One study reported blinding of the provider, patients and lab [25], whereas 3 studies did not blind patients [31, 33, 35], one study blinded patients only [32] and one study did not describe methods to blind adequately [34]. Table 3 reports the risk of bias summary and Table 4 reports the risk of bias graph for the RCTs.

**Table 3:**
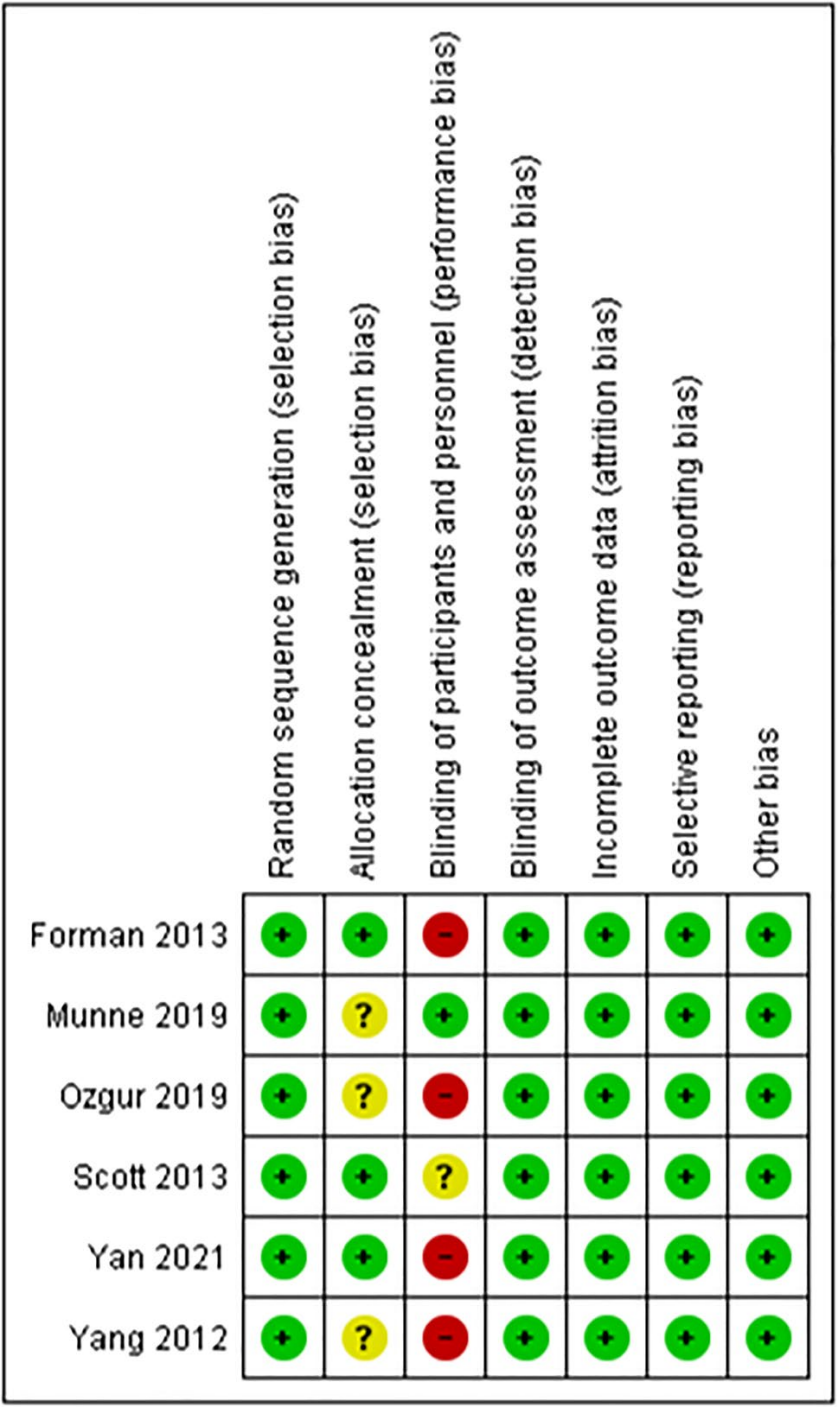
Risk of bias summary for RCTs

**Table 4:**
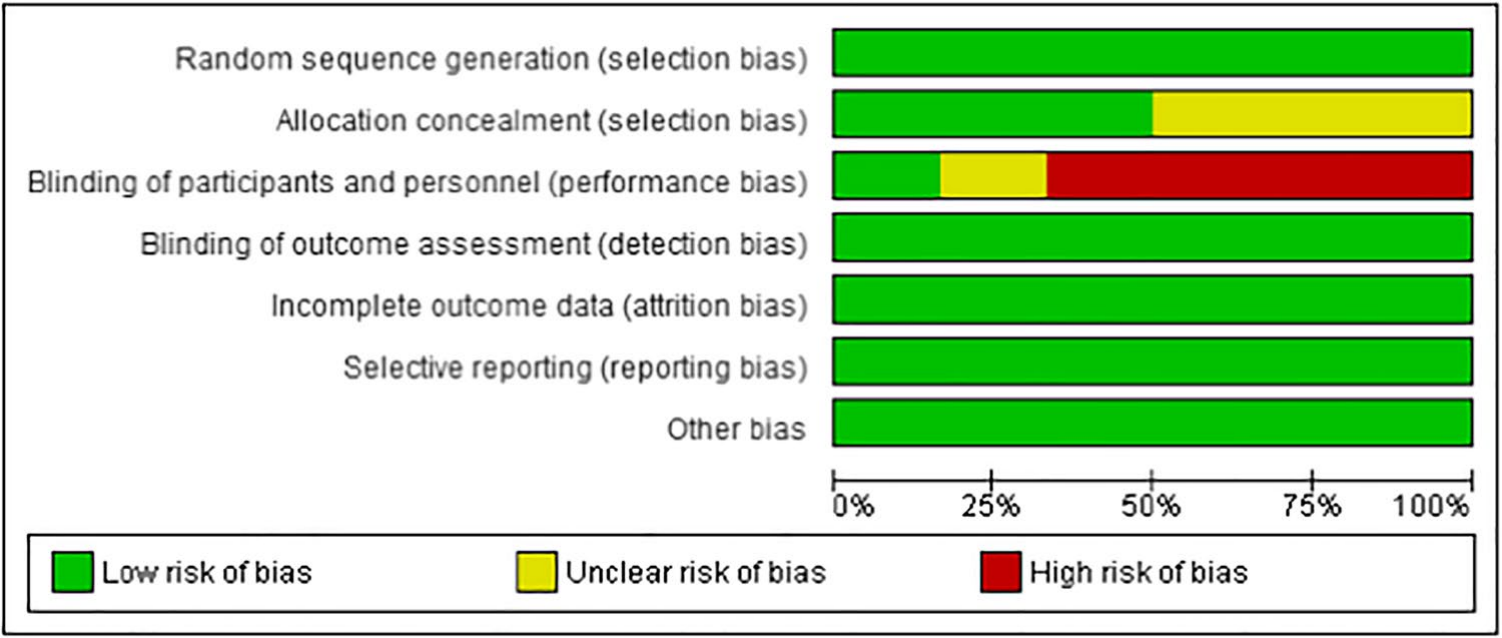
Risk of bias graph for RCTs

The 10 cohort studies included in the present meta-analysis had a NOS score between 6 and 9 (median 7). All studies described the selection of patients in both the PGT-A and control group. Only two studies matched the PGT-A group to a suitable control group prior to analysis [30, 36], which may raise concerns about comparability bias in the remaining studies. The primary outcome was well-defined in all studies with sufficient follow-up. Table 5 reports the risk of bias for all cohort studies.

**Table 5:** Newcastle–Ottawa Quality Assessment Scale for cohort studies

Studies	Selection				Comparability	Outcome			Total score
	Representativeness of the exposed cohort	Selection of non-exposed cohort	Ascertainment of exposure	Demonstration that outcome of interest was not present at start of study	Comparability of cohorts on the basis of the design analysis	Assessment of outcome	Was follow up long enough for outcomes to occur	Adequacy of follow up of cohorts	
Schoolcraft (2010)		*	*	*	**	*	*	*	8/9
Forman (2012)		*	*	*		*	*	*	6/9
Schoolcraft (2013)		*	*	*	*	*	*	*	7/9
Lee (2015)		*	*	*	**	*	*	*	8/9
Kang (2016)		*	*	*		*	*	*	6/7
Whitney (2016)		*	*	*	*	*	*	*	7/9
Coates (2017)		*	*	*	*	*	*	*	7/9
Liss (2018)		*	*	*	**	*	*	*	8/9
Lee (2019)		*	*	*		*	*	*	6/9
Sato (2019)		*	*	*	**	*	*	*	8/9

### Composite outcome live birth rate and ongoing pregnancy per embryo transfer

**Fig. 2:**
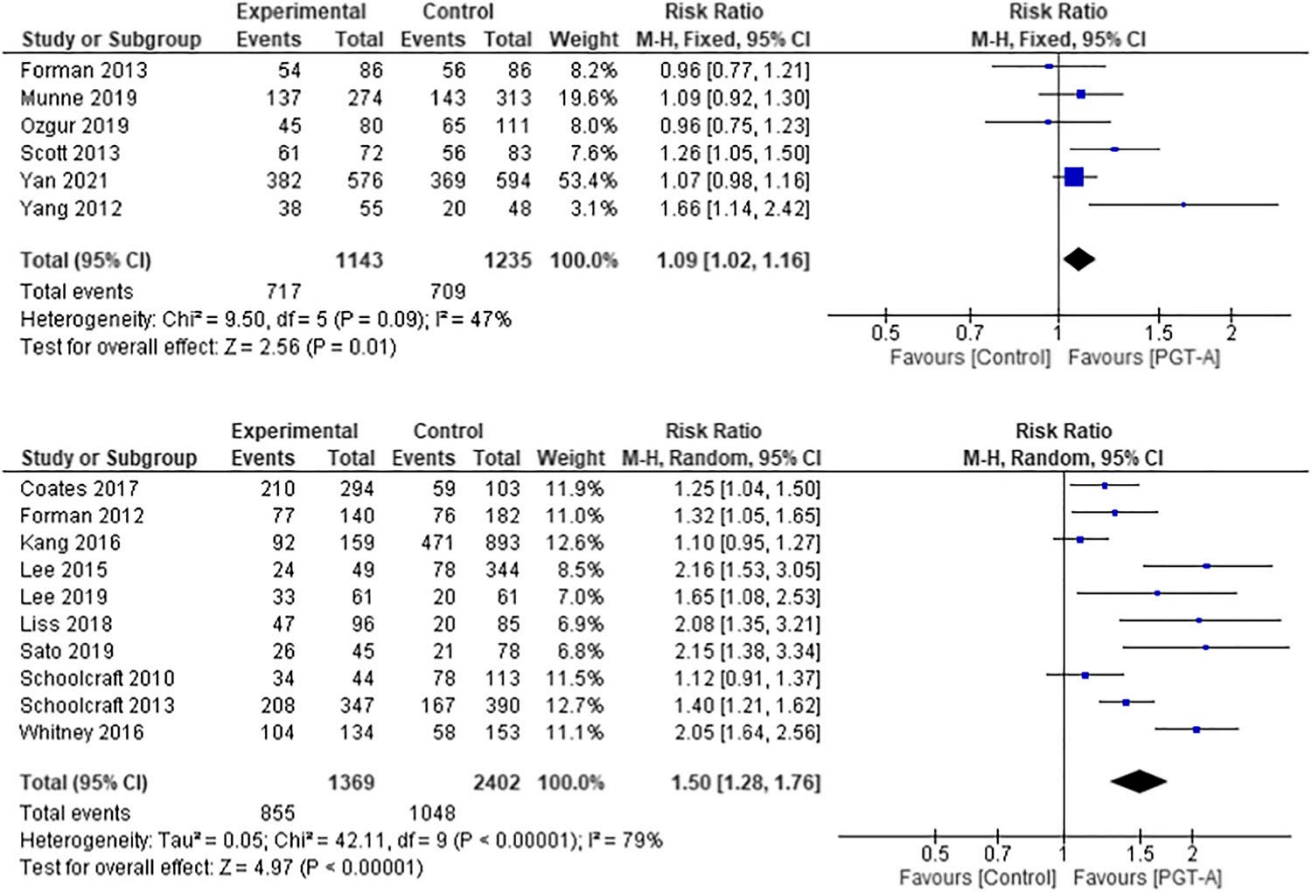
Forest plots showing the results of the meta-analysis on composite outcome of live birth rate and ongoing pregnancy per embryo transfer, comparing the effect of traditional morphological methods (control) and CCS based PGT-A

All studies included in this meta-analysis provided details of LBR or OPR as their primary outcome. There was variation in the definition of ongoing pregnancy amongst the studies, with some using a foetal heart ≥ 20 weeks and others ≥ 24 weeks. Given the stillbirth rate is very low, it is highly likely that ongoing pregnancies ≥ 20 weeks proceed to live births, as demonstrated by one of the largest RCTs in this meta-analysis [25]. As highlighted in Fig. 2, the PGT-A group had higher pooled LBR/OPR per embryo transfer in both the RCT (RR 1.09, 95% CI 1.02–1.16; *p* = 0.01) and cohort studies (RR 1.50, 95% CI 1.28–1.76; *p* < 0.001).

### Live birth rate per embryo transfer

**Fig. 3:**
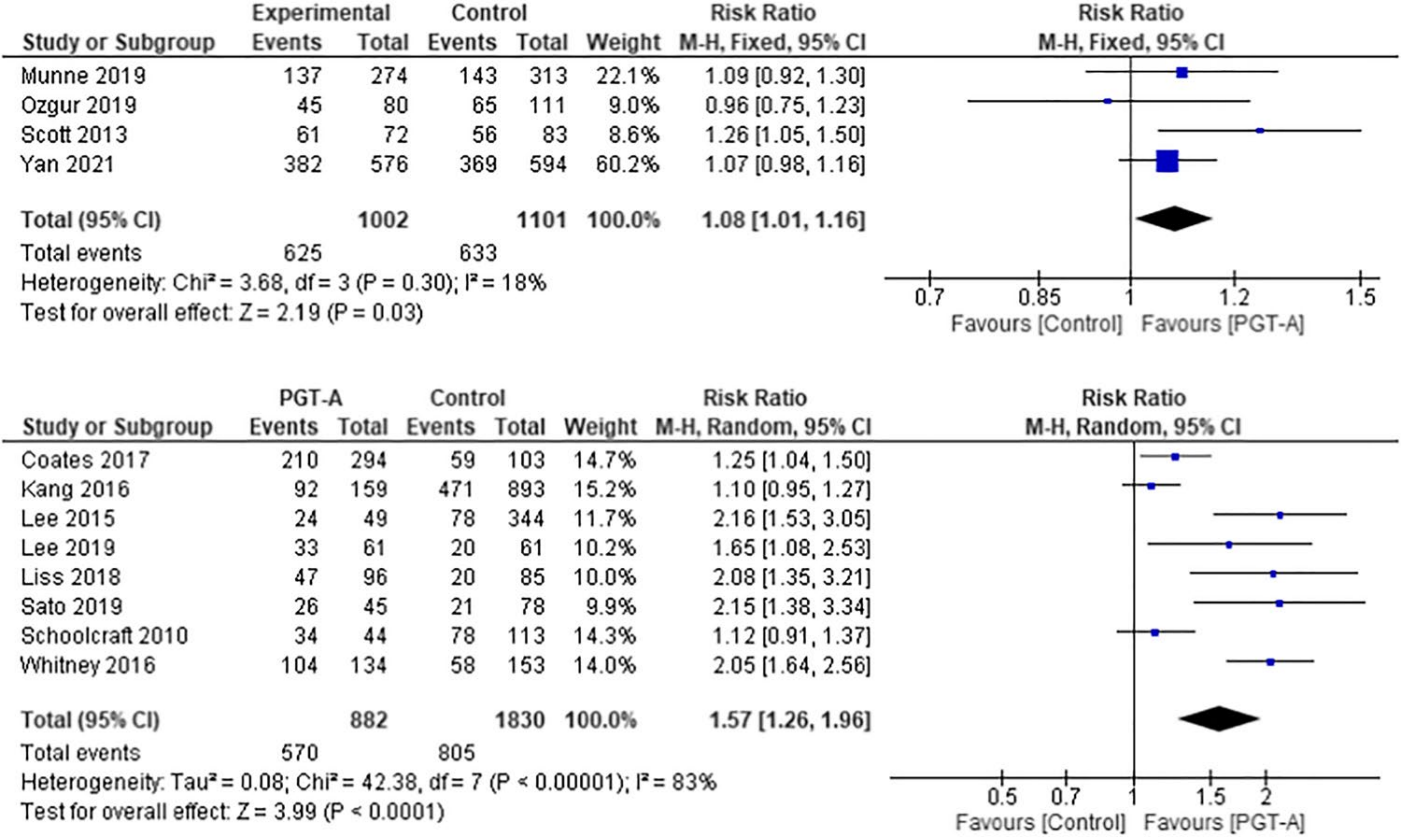
Forest plots showing the results of the meta-analysis on live birth rate per embryo transfer, comparing the effect of traditional morphological methods (control) and CCS based PGT-A

Twelve studies [25, 28–31, 34–36, 38–41] provided details on the LBR per embryo transfer (Fig. 3).

In one study, the live births plus sustained pregnancies could not be differentiated from the OPR figure [27]. Ongoing pregnancy was defined as an ongoing pregnancy > 24-weeks gestation. Whilst most pregnancies over 24 weeks result in a live birth, there may be a small number of stillbirths, which may alter the accuracy of the results. For this reason, the study by Forman et al. was included only in the composite outcome live birth rate and ongoing pregnancy per embryo transfer analysis and not in the live birth rate per embryo transfer analysis.

The benefit of using PGT-A to improve live births in the RCTs (RR 1.08, 95% CI 1.01–1.16) was statistically significant (*p* = 0.03). In the cohort studies, the benefit was also demonstrated (RR 1.57, 95% CI 1.26–1.96; *p* < 0.001).

### Ongoing pregnancy rate per embryo transfer

**Fig. 4:**
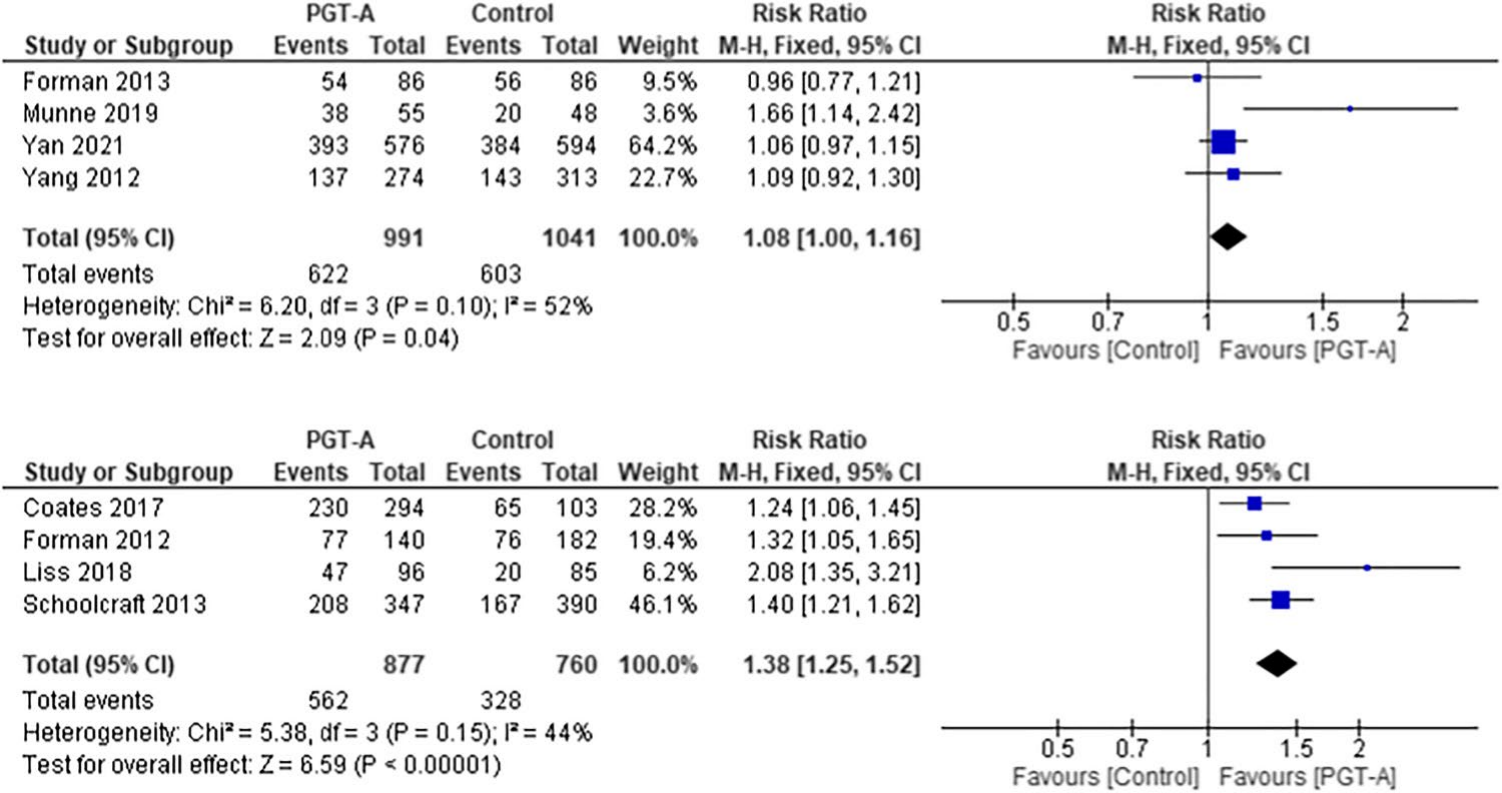
Forest plots showing the results of the meta-analysis on ongoing pregnancy rate per embryo transfer, comparing the effect of traditional morphological methods (control) and CCS based PGT-A

Over half the studies (*n* = 8) used OPR as their primary end outcome, including 4 RCTs [25, 32, 33] [31] and 4 cohort studies [27, 37, 39, 40] (Fig. 4). Two RCTs reported both LBR and OPR and therefore appear in both sub analyses [25, 31]. It should be noted that the STAR study was an intention-to-treat study, and the data used in the analysis reflects the actual intervention provided [25].

The pooled OPR per embryo transfer was higher in the PGT-A group compared to the control in the RCTs (RR 1.08, 95% CI 1.00–1.16; *p* = 0.04). This benefit was also considered statistically significant in the cohort studies (RR 1.38, 95% CI 1.25–1.52; *p* < 0.001).

### Implantation rate

**Fig. 5:**
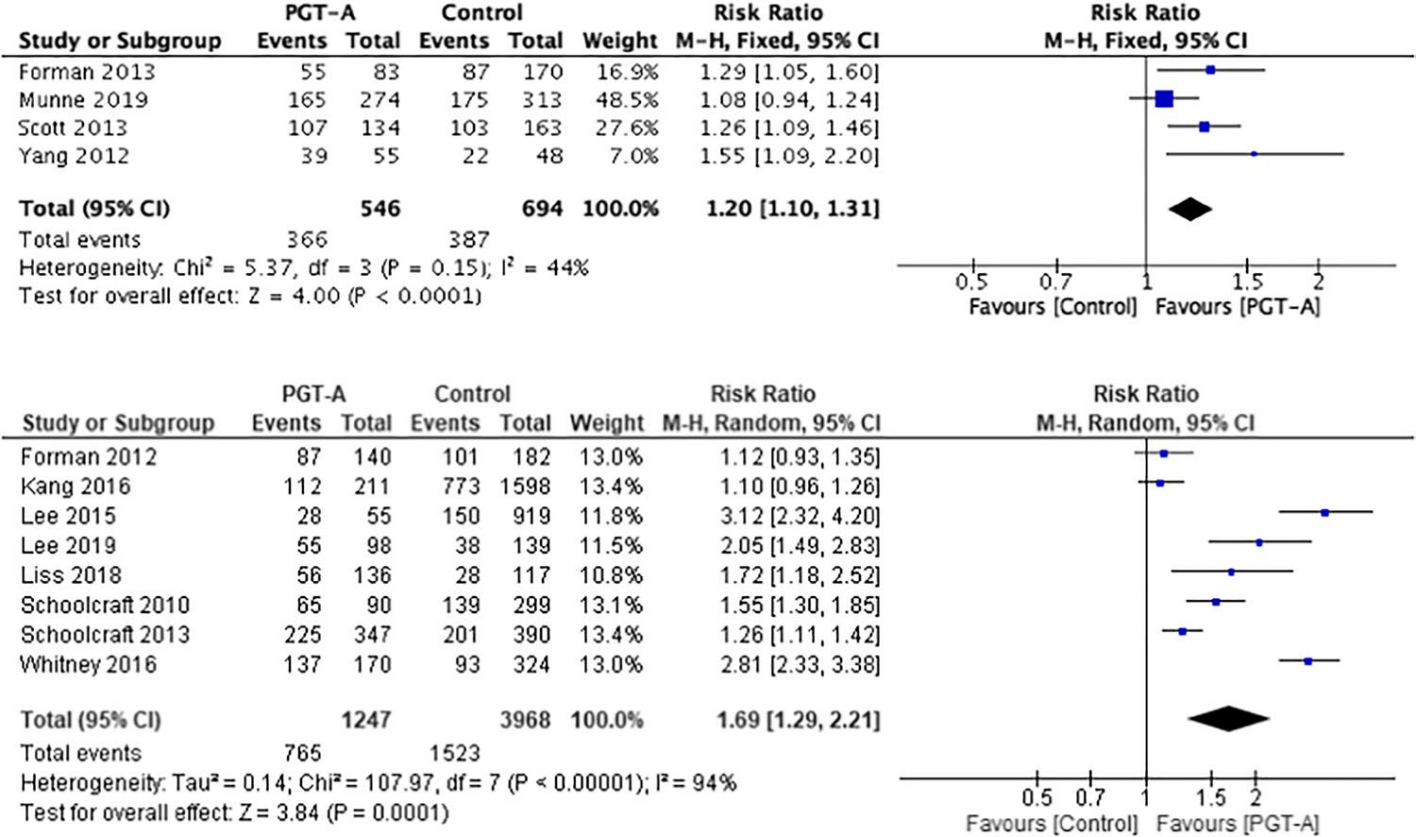
Forest plots showing the results of the meta-analysis on implantation rate, comparing the effect of traditional morphological methods (control) and CCS based PGT-A

The majority of studies (*n* = 12) reported IR or provided clinical pregnancy data and the number of embryos transferred (Fig. 5) [25, 27–29, 32–34, 36–38, 40, 41].

Studies excluded from this analysis were those where only LB per embryo transferred was reported [39], or where no data was available [30, 35]. Both the pooled results of the RCTs (RR 1.20, 95% CI 1.10–1.31; *p* < 0.001) and the cohort studies (RR 1.69, 95% CI 1.29–2.21; *p* < 0.001) showed a statistically significant higher IR in the PGT-A group compared to the control group.

### Miscarriage rate per clinical pregnancy

**Fig. 6:**
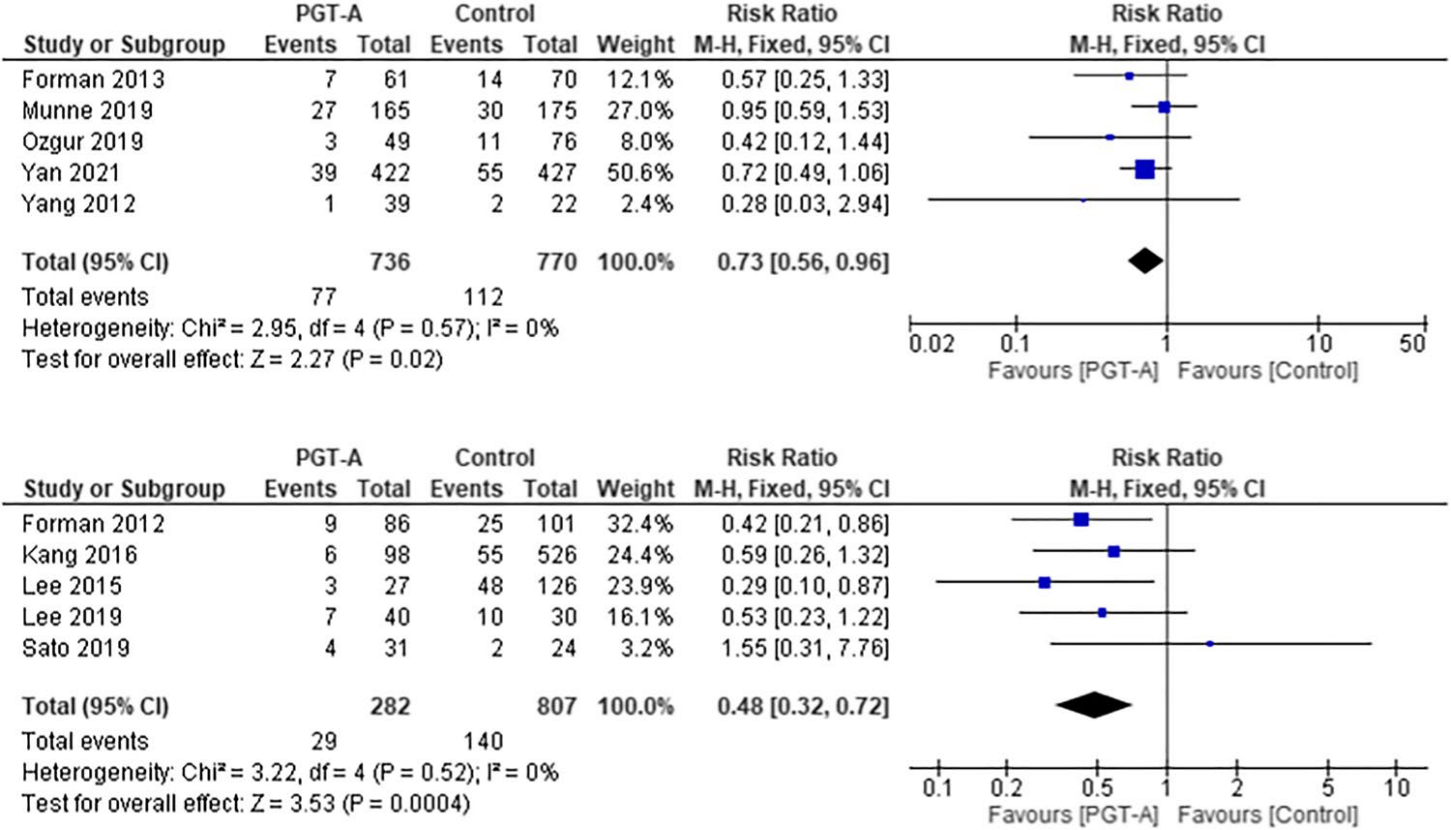
Forest plots showing the results of the meta-analysis on miscarriage rate per clinical pregnancy, comparing the effect of traditional morphological methods (control) and CCS based PGT-A

Five RCTs [25, 31–33, 35] and 5 cohort studies [27, 29, 30, 38, 41] evaluated miscarriage as an outcome (Fig. 6).

Three studies were excluded from this analysis because the data was either conflicting [28, 37], incomplete or the definition of missed abortion was not clearly defined [37, 40]. The pooled RCT data showed a trend for higher miscarriage rate in the control group, which was statistically significant (RR 0.73, 95% CI 0.56–0.96; *p* = 0.02). The pooled analysis of the cohort studies (*n* = 1089) also demonstrated a statistically significant higher miscarriage rate in the control group (RR 0.48, 95% CI 0.32–0.72; *p* = 0.0004).

### Miscarriage rate per embryo transfer

**Fig. 7:**
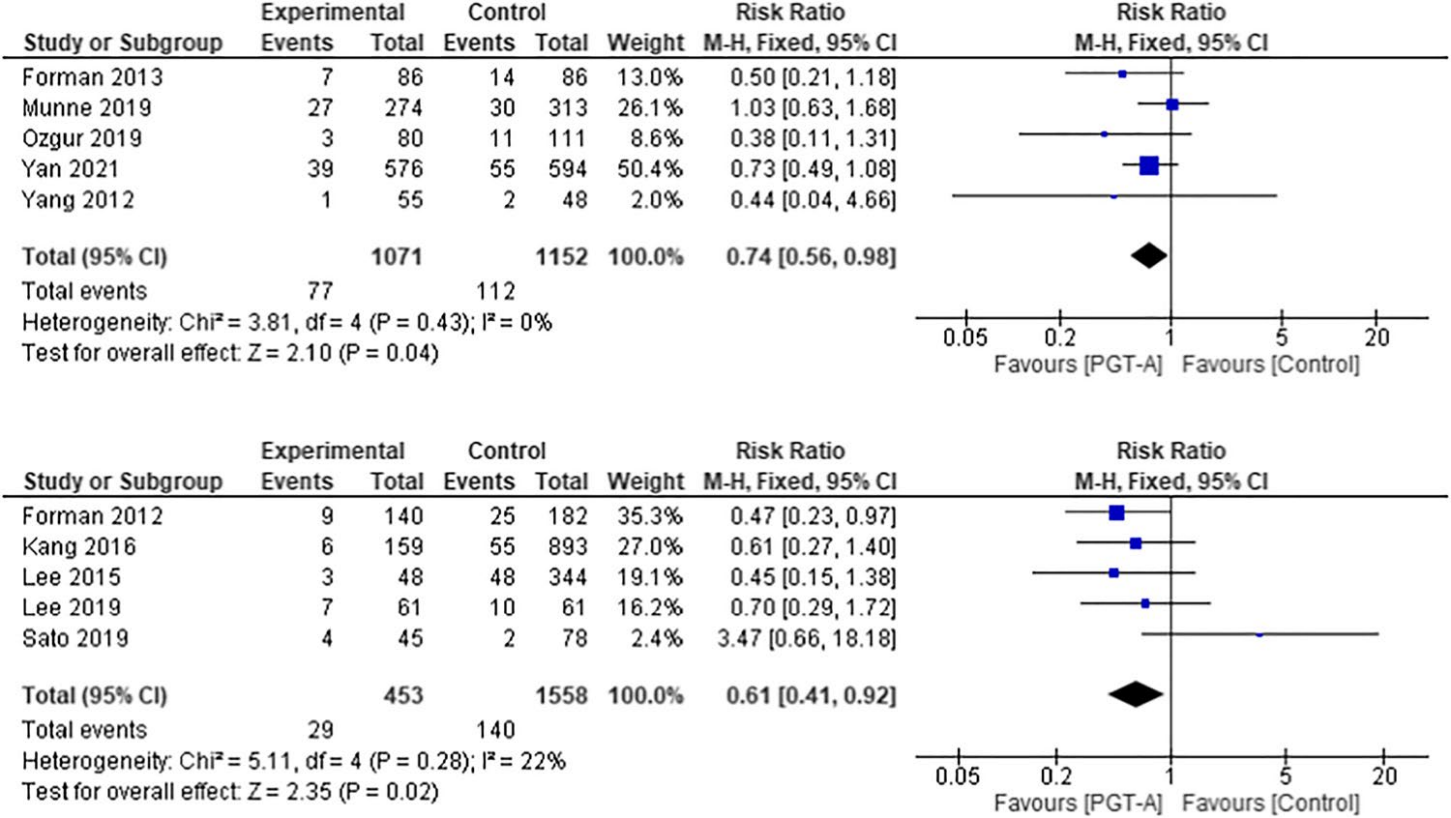
Forest plots showing the results of the meta-analysis on miscarriage rate per embryo transfer, comparing the effect of traditional morphological methods (control) and CCS based PGT-A

The miscarriage rate was analysed per embryo transfer amongst 10 studies (Fig. 7).

The pooled analysis for the RCT studies [25, 31–33, 35] showed a statistically significant trend for a higher miscarriage rate in the control group (RR 0.74, 95% CI 0.56–0.98; *p* = 0.04), as well as in the pooled analysis of cohort studies [27, 29, 30, 38, 41] (RR 0.61, 95% CI 0.41–0.92; *p* = 0.02).

### Multiple pregnancy rate

**Fig. 8:**
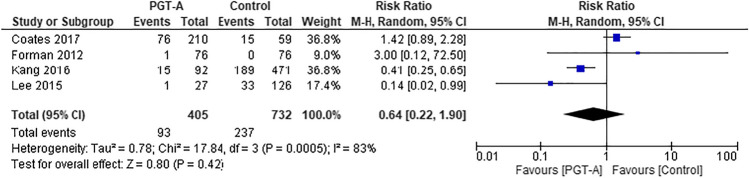
Forest plots showing the results of the meta-analysis on multiple pregnancy, comparing the effect of traditional morphological methods (control) and CCS based PGT-A

Only four cohort studies reported data concerning multiple pregnancy rates (Fig. 8) [27, 29, 38, 39]. Although the study by Coates et al. is included in this analysis, it is important to acknowledge that the numbers provided represent twin live birth rates and not multiple clinical pregnancy rates [39].

Two RCTs in particular were not included in this analysis for the following reasons. The first used a single untested blastocyst for transfer in the control group [31] and the second used two embryos for transfer [33]. Amongst the pooled cohort studies, higher multiple pregnancy rates were observed in the control compared to PGT-A groups, but this did not reach statistical significance (RR 0.64, 95% CI 0.22–1.90; *p* = 0.42).

### PGT-A for specific indications

Two studies specifically analysed outcomes in women with either RIF or RM [30, 41]. As the study by Lee et al. [41] did not include a control group, a pooled analysis was not feasible.

AMA was commonly cited as an indication for PGT-A, with several cohort studies evaluating its effect on LBR and or OPR. However, studies are not consistent with regard to age stratification using the following: < 35 vs ≥ 35 [25], < 38 vs ≥ 38 [29, 41] and more complex stratifications such as the following: ≤ 34, 35–37, 38–40, 41–42 and ≥ 43 [27, 28]. As such, the variation in age classification makes the pooling of results impractical.

### Heterogeneity analysis

The *I*^2^ test result for heterogeneity of the pooled risk estimates varied from 0 to 94% amongst the cohort and RCTs. The heterogeneity was overall higher amongst the cohort studies.

## Discussion

To the best of our knowledge, this is the first meta-analysis to systematically review all studies comparing CCS-based trophectoderm biopsy PGT-A on blastocysts only to a control group.

We demonstrate herein that CCS-based PGT-A is associated with a statistically significant higher LBR per embryo transfer and OPR per embryo transfer in both the RCT and cohort studies. Conversely, however, a previously published systematic review assessing the use of FISH to screen an arbitrary number of chromosomes, and, or, use cleavage-stage biopsies, failed to demonstrate similar improvements [12]. However, it should be acknowledged that the primary outcome measure in the aforementioned study was LBR per woman following PGT-A and not per embryo transfer.

Previously published systematic reviews which have not demonstrated improvements in LBR may be attributed to the fact that the majority of studies included in the analyses assessed earlier methods of PGT-A, with significant discrepancies between the techniques for genetic testing implemented between studies. Reproductive outcomes following the implementation of CCS PGT-A have improved significantly as technology has evolved, and therefore, it is inappropriate to combine earlier outcomes with those following the use of recent newer techniques. Consequently, our findings are consistent with a more recent meta-analysis focussing on modern CCS-based techniques, including both cleavage and blastocyst stage biopsies, whereby increased clinical pregnancy rate, OPR and LBR per cycle associated with PGT-A were also demonstrated [42].

Amongst studies whereby improved LBR with PGT-A are not recognised benefits, the success of PGT-A was deemed to be age-dependent. This is exemplified by one study whereby improved rates with PGT-A were only observed in women above 35 years old [43]. This has also been demonstrated in various studies, whereby there were higher OPR observed not only in young women < 35 (*p* = 0.01), but also in women > 40 years using PGT-A (*p* = 0.03) compared to controls [27, 28]. Similarly, in the Whitney et al. study, the LBR per embryo transfer was higher in women aged between 38 and 42 years old (*p* = 0.01) receiving PGT-A [28]. Evidently, the overall benefit of PGT-A is overshadowed by the favourable outcomes demonstrated in young healthy couples, and where success is only deemed significant in high-risk groups, such as AMA and RIF [44].

It is also important to acknowledge that the relative risk for the composite ongoing pregnancy and live birth rates amongst the RCT and cohort studies was 1.09 and 1.50 respectively. This may not be considered a significant improvement, considering the potential risk of loss of embryos following biopsy between the studies, lack of euploidy and the additional expense of the procedures. It is important therefore for clinicians to effectively counsel couples regarding the risks and benefits of PGT-A in a non-biased manner, whilst considering the patients’ priorities and the variation of risks between clinics.

The current meta-analysis also demonstrates a significant increase in IR when using CCS-based PGT-A. This is in keeping with two systematic reviews, consisting of three RCTs [45, 46]. It is well established that blastocysts have a higher implantation rate than cleavage-stage embryos because of the ability to aspirate more cells [47]. It has been argued that blastocyst biopsies contain a comparatively higher content of DNA templates compared to the cleavage stage, which is believed to improve the sensitivity and specificity of PGS [46]. As such, the cells sampled from the trophectoderm can accurately predict the chromosome complement of the inner cell mass and are therefore less vulnerable to mosaicism [48], with notably statistically significant increased LBRs per embryo transfer [43]. Conversely, cleavage-stage biopsy is associated with increased traumatic injury and a 39% reduction in implantation rate [49]. Thus, given that the studies included in this meta-analysis assessed trophectoderm biopsies from blastocysts only, this provides further evidence for these positive outcomes.

CCS-based PGT-A was also found to significantly reduce the miscarriage rate per clinical pregnancy and per embryo transfer in both the cohort studies and RCTs. It should be acknowledged however, that the causes of miscarriage are multifactorial, and therefore, in order to interpret the relationship between miscarriage rate and the use of PGT-A, significantly larger sample sizes are required for analysis in order to generalise conclusions.

We also demonstrate lower multiple pregnancies following PGT-A compared to the control group amongst the cohort studies only. Although DET is associated with improved LBR [1], the increased risk of multiple gestations along with maternal and neonatal complications has led to a trend in undertaking SET, using the highest quality embryo, facilitated by the use of PGT-A. Although the mean number of embryos transferred per patient was higher in the control groups, only 4 studies used SET exclusively in both arms of the study [27, 29, 38, 39]. Amongst these studies, one reported the multiple pregnancy rate, in which there was one case of multiple pregnancies described in both arms [27]. This is in keeping with the low rates of multiple pregnancies associated with SET [39]. Given the improvement in implantation rate observed with PGT-A therefore, it is perhaps feasible to recommend single euploid embryo transfer in women undergoing IVF as standard of care, in order to overcome the associated risks of multiple pregnancies.

Most studies did not undertake PGT-A for a specific indication and presented findings from women with a good prognosis. For example, the RCT from the STAR Study Group included women aged between 25 and 40 years old [25]. In addition, they excluded cases with diminished ovarian reserve, more than two failed IVF cycles and more than one miscarriage or severe oligospermia. The two studies that did evaluate RM and LBRs per embryo transfer found a benefit with PGT-A; however, it was not possible to deduce the overall effect in this meta-analysis due to a lack of control groups for comparison. With regard to RIF, one study deduced that the LBR per embryo transfer almost doubled with CCS-based PGT-A [30]. Contrariwise, such relationships were not observed amongst studies using FISH on cleavage stage embryos [50, 51].

In previous practice, embryos deemed to be abnormal due to mosaic chromosomal losses and gains were excluded from transfer [25], which potentially reduced the overall chances of livebirth, as less blastocysts were available for transfer. In a recent study assessing live births following the transfer of chromosomally *abnormal* embryos after PGT-A, it was apparent that the percentage of all estimated cycles transferring abnormal embryos differed substantially between centres worldwide, including Europe, Asia and USA and Canada combined, with rates of 7%, 11.6% and 67.4% reported respectively [52]. Thus, the percentage of abnormal embryos identified from PGT-A varies significantly between clinics internationally. Furthermore, recent evidence suggests approximately 41% of mosaic embryos transferred were associated with ongoing implantation [53], and indeed, a small proportion of such embryos may be viable and consequently achieve a live birth [25, 54–56]. This is supported by evidence from a recent double-blinded prospective non-selection trial which demonstrated similar rates of live birth and miscarriage across 484 euploid, 282 low-grade mosaic and 131 medium-grade mosaic embryos [57]. Given that obstetric and neonatal outcomes were also similar between study groups, this suggests that low–medium mosaic embryos have the same potential to develop as fully euploid ones, mostly because the mosaicism in the trophectoderm occurs after the trophectoderm and inner cell mass differentiation [57]. In Sato’s study, 5 patients out of 41 receiving PGT-A only had euploid embryos with suspicion of mosaicism available. These were transferred and resulted in 3 live births [30]. More recently, non-invasive methods to analyse the genetics of the embryo using embryonic cell–free DNA released into the culture media have been proposed as an alternative to the current invasive testing of the embryo. Although concordance rates between current methods of PGT-A and newer non-invasive methods (niPGT-A) are variable however, the latter are associated with promising results [58].

### Strengths and limitations

Despite a large number of studies assessing if CCS-based PGT-A improves IVF outcomes, due to the heterogeneous number of outcomes used (delivery rate, LBR, clinical pregnancy rate, biochemical pregnancy rate, IR, OPR) and differences in definitions of each outcome measure, many studies did not fulfil the inclusion criteria for this meta-analysis. There was also variation in the number of embryos transferred and whether fresh or frozen embryos were used. It could be argued however that the composite primary outcome of OPR and LBR utilised herein, facilitated the inclusion of greater numbers of studies within this meta-analysis, whilst specifically assessing blastocyst biopsy, resulting in more robust and focused findings.

Many authors have criticised studies using a primary outcome measure per embryo transferred to determine the effects of PGT-A. This is based on the argument that embryos selected by PGT-A have a higher potential for successful implantation, but the process itself results in fewer embryos selected for transfer. Given that women who have aneuploid or unsuitable embryos identified by PGT-A do not undergo embryo transfer, if a study only reports on the women having an ET or on outcomes per embryo transfer, there is a resulting bias in the study design, in favour of PGT-A [11]. This is because by default, all women who do not have an ET and therefore do not get pregnant are excluded from the analysis. It has been argued that in order to draw fairer comparisons, all embryos should be considered, including treatments that could have had PGT-A, such that treatment outcomes should be calculated per woman (including all women going for treatment), or per started treatment cycle, which is reflected by the cumulative LBR, as this would determine whether the embryos not used following PGT-A were rightfully excluded [11]. This would allow for a more accurate interpretation of the overall benefit, or harm, following PGT-A.

It has also been proposed that due to significant bias in the design of previously published studies, what can be deduced from the evidence so far is that PGT-A is merely effective in differentiating viable from non-viable embryos, as supported by superior LBR and ongoing pregnancy rates demonstrated per embryo transfer, as the euploid embryos transferred are more likely to implant, resulting in successful clinical pregnancy outcomes due to the reduced likelihood of altered chromosomal material resulting in an adverse outcome [19]. Whilst we acknowledge that OPR and LBR outcome per cycle was not assessed in this meta-analysis, this was because our primary intention was to investigate the impact of PGT-A on OPR and LBR per embryo transfer.

Moreover, it is well recognised that both the timing of the biopsy and the type of chromosomal screening implemented impact outcomes [59]. Due to a relative paucity of studies, all newer forms of CCS were considered in this meta-analysis with only blastocyst-stage biopsies performed. Live birth rates per embryo transfer were significantly increased amongst day 5 biopsy groups when outcomes from days 3 and 5 biopsies have been compared [49]. Our own statistically significant findings, therefore, may be attributed to the nature of positive outcomes associated with day 5 biopsies. Furthermore, recent studies have compared different types of CCS methods, whereby NGS has been shown to be effective at detecting whole and segmental aneuploidies and demonstrated an improvement in pregnancy outcomes compared to other forms of CCS [60]. This supports our own findings, especially as the majority of studies included in the meta-analysis employed NGS CCS exclusively. With the widespread use of NGS, future studies are required to investigate the specific benefit of PGT-A, as well as the potential for harm, when only using NGS for CCS.

Furthermore, it should be acknowledged that heterogeneity was higher amongst the cohort studies (0–94%) compared to the RCTs (0–52%), despite the statistical significance demonstrated. It is important therefore to consider variation in the results between studies when drawing overall conclusions of the effects of PGT-A on the outcomes described. This also reflects the demand for further high-quality standardised RCTs assessing the effect of PGT-A, such that conclusions drawn can then be generalisable. This is particularly important considering the contradictory evidence so far regarding PGT-A and the need to improve the overall understanding of the outcomes.

## Conclusion

In conclusion, this study showed an overall improvement in the composite outcome of live birth and ongoing pregnancy per embryo transfer between CCS-based PGT-A at the blastocyst stage on day 5 embryos, compared to quality assessment using morphology alone. In addition, PGT-A was associated with an improvement in implantation rate and a reduction in miscarriage rate. It is essential that future studies evaluating these newer CCS techniques use rigorous standardised approaches and outcome metrics to facilitate appropriate comparisons.

## Abbreviations

IVF: In vitro fertilisation
ART: Assisted reproductive technologies
PGT-A: Pre-implantation genetic testing for aneuploidy
PGS: Pre-implantation genetic screening
FISH: Fluorescence in-situ hybridisation
CCS: Comprehensive chromosomal screening
CGH: Array comparative genomic hybridisation
SNP: Single nucleotide polymorphism
qPCR: Quantitative polymerase chain reaction
NGS: Next-generation sequencing
LBR: Live birth rate per embryo transfer
OPR: Ongoing pregnancy rate per embryo transfer
IR: Implantation rate
RCT: Randomised control trial
